# Item format statistics and readability of extended matching questions as an effective tool to assess medical students

**DOI:** 10.1038/s41598-022-25481-y

**Published:** 2022-12-05

**Authors:** Anna Frey, Tobias Leutritz, Joy Backhaus, Alexander Hörnlein, Sarah König

**Affiliations:** 1 Department of Internal Medicine I, University Hospital of Würzburg, Oberdürrbacher Str. 6-8, 97080 Würzburg, Germany; 2 Institute of Medical Teaching and Medical Education Research, University Hospital of Würzburg, Würzburg, Germany; 3 University Datacenter, University of Würzburg, Würzburg, Germany

**Keywords:** Health occupations, Medical research

## Abstract

Testing based on multiple choice questions (MCQ) is one of the most established forms of assessment, not only in the medical field. Extended matching questions (EMQ) represent a specific type of MCQ designed to require higher levels of cognition, such as problem-solving. The purpose of this evaluation was to assess the suitability and efficiency of EMQ as an assessment method. EMQ were incorporated into the end-of-semester examination in internal medicine, in which 154 students participated, and compared with three established MCQ types. Item and examination quality were investigated, as well as readability and processing time. EMQ were slightly more difficult to score; however, both item discrimination and discrimination index were higher when compared to other item types. EMQ were found to be significantly longer and required more processing time, but readability was improved. Students judged EMQ as clearly challenging, but attributed significantly higher clinical relevance when compared to established MCQ formats. Using the Spearman-Brown prediction, only ten EMQ items would be needed to reproduce the Cronbach’s alpha value of 0.75 attained for the overall examination. EMQ proved to be both efficient and suitable when assessing medical students, demonstrating powerful characteristics of reliability. Their expanded use in favor of common MCQ could save examination time without losing out on statistical quality.

## Introduction

Assessment can provide educators with feedback on the quality of their teaching, areas for improvement, and whether learning outcomes have been achieved. The type of written test instrument best suited to assess knowledge in medical students is highly contested. Testing based on multiple-choice questions (MCQ) isone of the most established and successful forms of assessment^1^. An outstanding feature thereof is the ability to conduct an examination for a great number of students, and large sample sizes guarantee objective scoring. However, the development of high-quality multiple-choice test items is an elaborate process^2^. Not only has factual knowledge to be measured, but also higher cognitive abilities such as problem solving, synthesis, and evaluation skills. Well-written MCQ require students to apply their knowledge to clinical case scenarios. Different concepts of creating questions and answers have been developed to permit the measurement of different types of knowledge^3^. The single best answer (SBA) format is the most frequent in medical education. In items defined as multiple correct answer questions (Pick-N), students need to choose more than one best option from a list^4^. Multiple true–false questions (MTF) contain several options, and each of them has to be considered independently^5^.

Extended-matching questions (EMQ) are based on a list of possible answers (diagnoses, drugs, etc.). Several short patient scenarios are presented, and students have to match the most appropriate answer from the list. From the outset, EMQ are designed to assess higher cognitive levels of thinking and are strongly recommended for testing advanced medical students or in high-stakes examinations^6^. It has to be pointed out that EMQ have already been determined as suitable to assess problem-solving and specifically diagnostic skills^7^. Furthermore, they proved their ability to monitor the processes and development of clinical reasoning in a study comparing the performance of final-year students to fourth/fifth-year resident doctors^8^.

There is a paucity of prospective studies within this area in the current literature in which the performance of EMQ and other MCQ types are tested on a single cohort. The aim of this study was to explore the suitability, item quality, and resource effectiveness of EMQ, the goal being to give educators the necessary confidence during the design phase when constructing written assessments for students of medicine.

## Methods

### Study design and participants

The prospective study took place within the framework of a compulsory and real examination at the medical school of the Julius-Maximilians-University of Würzburg in Germany, which offers a six-year curriculum towards the degree of medicine. Students were taught the subject of internal medicine in the sixth and seventh semesters. Teaching activities included lecture series, seminars, and small-group bedside teaching with patients. The data for the study were collected in the winter semester of 2019/2020 in two ways: (1) Academic achievement of 154 students as fourth year medical undergraduates (after completion of the seventh semester) was assessed through a tablet-based digital examination using CaseTrain, a web application developed by the University of Würzburg^9^. (2) Students were requested to evaluate this examination in an anonymous online survey.

### Examination in internal medicine, question types, and scoring

The standard format of the examination in internal medicine includes eight sub-disciplines of internal medicine, and experts from each discipline contributed to altogether 60 MCQs (47 SBA, eight MTF and five Pick-N) as follows: endocrinology (ten questions), gastroenterology (six questions), hepatology (five questions), hemato-oncology (nine questions), pneumonology (ten questions), cardiology (nine questions), internal intensive care medicine (two questions), and nephrology (nine questions). MTF had four to five response options; true and false statements of the question type were evenly balanced over the entire examination. Pick-N comprised four to eight response options; the number of correct responses required was disclosed for each question. A total time of 115 min was scheduled for the examination. Each question had a value of one point; the maximum total score achievable in the examination was thus 60 points. SBA were valued at one or zero points for the correct or incorrect answer, respectively. Pick-N and MTF items allowed students to score half-points for partially correct answers. More than 50% of the options per item had to be selected correctly to score 0.5 points (e.g. three correct out of four or five response options).

We decided to integrate EMQ into this end-of-term examination to enhance the spectrum of test instruments. EMQ were developed in accordance with published examples^10^ and incorporated into the examination for the first time. An EMQ example is reproduced in the supplement (Supplemental Methods). The structure of the EMQ followed the Item Writing Manual of the National Board of Medical Examiners in the USA, published in 1998^11^. The layout of the CaseTrain examination player interface was adapted accordingly. The students received a demonstration version and were able to practice all MCQ types in advance. Four main patient complaints were selected for the EMQ to fit into the above mentioned sub-disciplines: dyspnea, thoracic pain, abdominal pain, and anemia. Each EMQ item had five clinical scenarios describing patients in a precise manner using key words and findings typical for their condition. The answer option lists encompassed eight diseases. Only one answer matched each scenario and each answer could be selected only once. Owing to the randomized composition of the examination, the EMQ were not necessarily consecutive. Students received one point for correctly matching all five pairs (scenarios and answer option), 0.5 points for three pairs, and zero points for fewer matches.

The examination pass mark was set at 60% (36 points), in accordance with the study regulations for the degree of medicine issued by the Faculty of Medicine. Standard German grades from 1 (excellent, ≥ 90% of the total score), 2 (good, ≥ 80%), 3 (satisfactory, ≥ 70%), 4 (sufficient, ≥ 60%) to 5 (insufficient = failed, < 60%) were set. For those passing the examination, students could be awarded a maximum of four bonus points for their correct answers to the EMQ and consequently improve their overall examination scores and thus potentially improve their grades. Therefore, a corrected total of 64 points was possible for the whole examination for successful candidates.

### Item statistics and examination quality

The item and examination quality were evaluated as follows. As *item difficulty* is an estimate of the skill level needed to pass an item^12^, optimal or moderate ranges were set between 0.4 and 0.8. *Item discrimination* is the ability of the item to differentiate between students with high and low performance in relation to the whole examination cohort. Based on Spearman’s correlation r´, the range of 0.2 ≤ r′ < 0.3 was considered acceptable, a value exceeding 0.3 as good^13^. The *discrimination index* indicates to which extent an item itself discriminates between students with higher and lower scores and was computed from equal-sized 27% quantiles of high and low-scoring groups of the examination cohort^14,15^. Acceptable values ranged between 0.2 and 0.3, good values between 0.3 and 0.4^16^. The *reliability* of a test refers to the internal consistency, which is applied as Cronbach’s alpha^17^, of which an acceptable value was defined as greater than 0.7^13,18^. In order to calculate the number of question types needed to achieve a certain Cronbach’s alpha for the whole examination, the predictive factor was determined using the Spearman-Brown formula^19^. This determines the number of replications required to achieve a pre-defined value of reliability. Therefore, the predictive factor for each question type was multiplied by the number of questions, aiming to predict Cronbach’s alpha for the whole examination. To compare the estimated duration of the predicted examination, the mean total processing time of each question type was multiplied by the predicted number of questions.

### Readability scores

Part-of-speech tagging was performed on the examination using TreeTagger as a tool for annotating text with part-of-speech and lemma information^20^. The number of words was assessed and compared across different question types. To enable an accurate evaluation of readability, short answer options, those not constituting a sentence and thus being captured quickly, were removed.

Type-token-ratio (TTR) is a score routinely used to define the complexity of language^21^. TTR documents the lexical richness or variety in vocabulary and is defined as the total number of unique words (= types/lemma) divided by the total number of words (tokens) in a given text segment. The closer the TTR ratio is to 1, the greater the lexical richness of the segment^22^. Text complexity usually decreases with text length^23^. To overcome the length dependency of TTR, we additionally calculated Yule’s K, which is regarded as invariant for text length^24^. Yule’s K is designed to measure the vocabulary richness of a text: the larger Yule's K, the less rich the vocabulary is.

### Log-file analyses of processing time

Response events during students’ processing were documented for each question by analysing the log files in CaseTrain. The time an item was opened for processing (opening), the first (initial response time), and final input (time to completion) were marked. We defined the time span from opening to the first input as the *read-in time*. The overall time spent on each item (from opening to completion) represented the *total processing time.*

### Students’ evaluation

On completion of the examination, students were asked to participate in an online survey using the platform EvaSys (version 8.0, Electric Paper Evaluationssysteme GmbH, Lüneburg, Germany). Students rated whether the question types were challenging and had valuable clinical relevance. The evaluation link was sent out to students by e-mail on the same day of the examination. Responses were recorded on a five-point Likert scale (1 = I do not agree at all, to 5 = I totally agree).

### Ethics and data protection

The institutional review board (IRB) of the University of Würzburg reviewed the project proposal and judged the project as not representing medical or epidemiological research on human subjects, thus not requiring ethics approval under German law. The IRB approved the project under the reference number 20220322 01. Furthermore and with respect to the rights of the student participants, the Faculty of Medicine Dean of Studies’ Office approved the examination data processing following the advice of the IRB, on the grounds that no personal data were retrieved and all results and performance data from the examination were anonymized prior to analysis. Thus, informed consent was not required for participation. Moreover, the survey run through the EvaSys platform was on a completely anonymous basis with a standard link to the platform for all participants. All data were processed and stored in total compliance with national and EU-wide data protection laws (BDSG-new, GDPR).

### Data analysis

Statistical analysis was conducted using IBM SPSS 27.0 (IBM SPSS, Armonk, New York, USA, RRID:SCR_019096) and R (version 4.0.1, R Foundation for Statistical Computing, Vienna, Austria, RRID:SCR_001905)^25^. R packages retrieved from CRAN snapshot 2021-04-01 were implemented. Descriptive information included item mean and standard deviation. The significance threshold was set to at least 0.05. Group differences were subjected to one-way ANOVA or Welch’s t-test^26^ as appropriate, depending on the result of Levene’s test for homogeneity of variance^27^. Post-hoc analysis was performed with Tukey’s honest significance test^28^. Cronbach’s alpha served as a measure of internal consistency and was calculated using the function reliability within SPSS.

## Results

### Students’ performance

In total, 154 students sat the MCQ examination. Four participants repeated the examination for the first time (2.6%). Nearly all (95.1%) of the students passed, attaining an average score of 45.09 ± 5.26 points. Student performance in the examination, as well as their academic achievement with and without EMQ bonus points are listed in Table 1. Grades from 1 to 5 were not normally distributed (Shapiro–Wilk-Test, p < 0.001).

**Table 1:** Student performance in the examination, achievement as standard results and following the addition of EMQ bonus points.

Grade	Points to be achieved in the ranges of the grades	Standard results, number (%)	Results with EMQ bonus, number (%)
1	54 to 60	6 (3.9%)	19 (12.3%)
2	48 to 53.5	43 (27.9%)	62 (40.3%)
3	42 to 47.5	72 (46.8%)	49 (31.8%)
4	36 to 41.5	27 (17.5%)	18 (11.7%)
5	≤ 35.5	6 (3.9%)	6 (3.9%)

The majority (97%) of the students took the opportunity to answer the EMQ, in addition to the 60 regular questions, with the aim of improving their overall results. The initial average grade was 2.90 ± 0.87, the addition of EMQ bonus points improved the average grade to 2.55 ± 0.98. A total of 53 (34%) participants were able to improve their achievement by one grade level.

### Quantitative analyses of the items and the examination

The overall item difficulty of the examination was considered as moderate, calculated as 0.75 ± 0.23 for the raw results without EMQ and 0.74 ± 0.23 following the addition of the EMQ bonus points. The mean item discrimination on examination level was acceptable with a value of 0.21 ± 0.11 from the outset and 0.22 ± 0.12 with EMQ, respectively.

**Figure 1:**
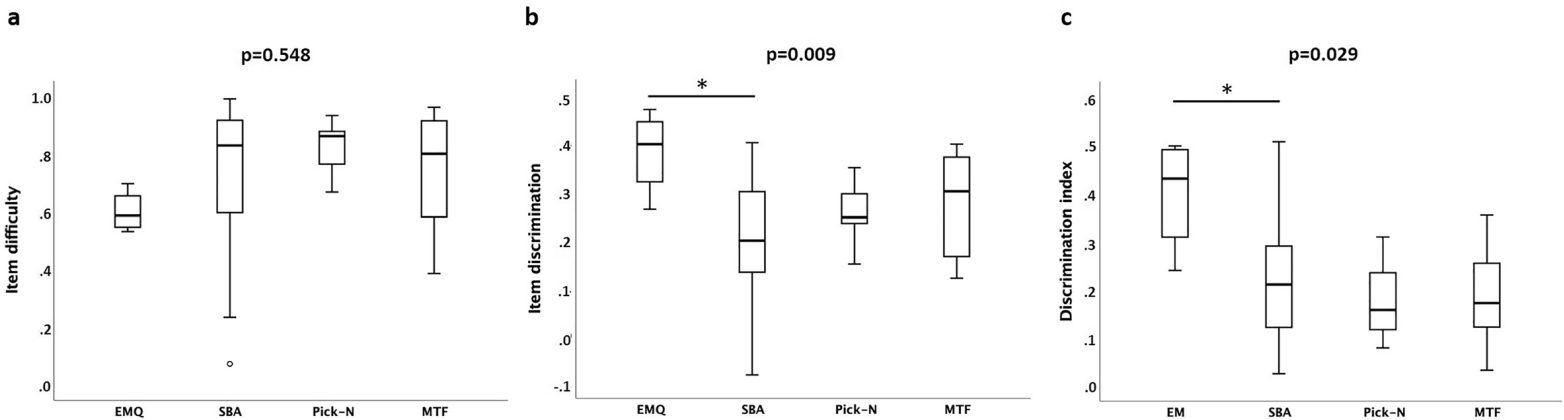
Items analyses of the MCQ types depicted by (**a**) item difficulty, (**b**) item discrimination, and (**c**) discrimination index. The differences between the types were analysed by one-way analysis of variance, *p < 0.05.

Item statistics of the different MCQ types are depicted in Table 2 and Fig. 1. There were no significant differences across the question types with respect to item difficulty. EMQ questions were the most difficult item, but still moderate with a calculated difficulty of 0.6 ± 0.07. The item discrimination of EMQ was the highest with a value of 0.38 ± 0.09 and considered good. However, item discrimination was heterogeneous across the question types. Of note, EMQ were revealed as significantly more discriminative than SBA (post hoc test). The mean discrimination index of the EMQ (0.40 ± 0.60) was also the highest among the question types, with EMQ being superior to SBA (post hoc test).

**Table 2:** Item characteristics of the different MCQ item types and results of the online survey.

Parameter	EMQ (n = 4)	SBA (n = 47)	Pick-N (n = 5)	MTF (n = 8)	p-value^#^
Item difficulty	0.60 (0.07)	0.75 (0.25)	0.82 (0.11)	0.74 (0.22)	0.548
Item discrimination	0.382 (0.0896)	0.199 (0.113)	0.254 (0.074)	0.286 (0.100)	0.009**
Discrimination index	0.400 (0.598)	0.216 (0.0189)	0.175 (0.048)	0.187 (0.056)	0.029*
Length of the question [word count]	149.3 (16.5)	74.3 (31.0)	69.8 (25.6)	52.0 (22.4)	< 0.001***
Type-token-ratio	0.61 (0.04)	0.75 (0.11)	0.72 (0.13)	0.81 (0.11)	0.032*
Yule’s K	142.7 (32.1)	170.2 (99.4)	187.9 (78.4)	128.0 (76.0)	0.589
Average read-in time [s]	47.7 (45.0)	30.9 (27.5)	26.4 (22.4)	22.3 (18.5)	0.054
Average processing time [s]	236.0 (111.0)	84.0 (63.7)	104.0 (57.5)	91.8 (61.2)	< 0.001***

Student rating	EMQ	SBA, MTF and Pick-N	p-value^#^		
“Question type was challenging”	3.16 (1.18)	2.71 (1.06)	0.003*		
“Question type had valuable clinical relevance”	3.79 (1.07)	3.09 (0.86)	< 0.001*		

Data are portrayed as mean (SD).

*p < 0.05; **p < 0.01; ***p < 0.001.

^#^As calculated by one-way ANOVA or Welch-ANOVA as applicable.

Cronbach’s alpha in the examination was calculated as 0.75 without and 0.77 with EMQ. The detailed reliability analyses and predictions are presented in Table 3. EMQ were the most powerful with only four questions reaching a Cronbach’s alpha of 0.58. In contrast, SBA needed as many as 47 questions to achieve an only slightly higher value of 0.66, however still not reaching the recommended threshold of 0.7 even. Following the application of the Spearman-Brown prediction formula to relate psychometric reliability to test length, only ten EMQ items were predicted to be necessary to reproduce the value of 0.75 as the overall reliability of the examination (without EMQ). In doing so, the duration of the hypothetical examination could thus be decreased to 40 min.

**Table 3:** Reliability analyses and prediction using the Spearman-Brown formula.

Parameter	EMQ (n = 4)	SBA (n = 47)	Pick-N (n = 5)	MTF (n = 8)
Cronbach’s alpha of the question type	0.58	0.66	0.29	0.45
Prediction of questions required to reach the examination reliability	10	83	41	33
Actual processing time [minutes] per question type, mean (SD)	3.93 (1.85)	1.40 (1.06)	1.73 (0.96)	1.53 (1.02)
Predicted processing time [minutes] based on the single question type	40	101	64	45

### Readability

**Figure 2:**
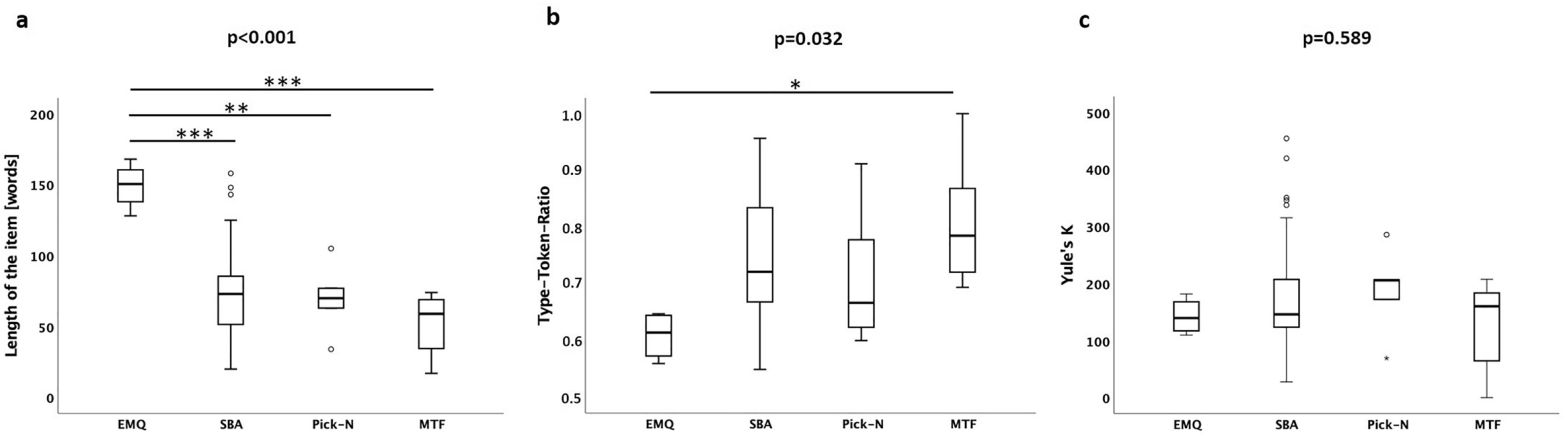
Readability as measured by (**a**) the length of the item, (**b**) TTR as degree of lexical variation, and (**c**) Yule’s K representing vocabulary richness. Parameters are depicted for each question type separately as box plots. *p < 0.05; **p < 0.01; ***p < 0.001.

The item text length varied significantly across the types (Table 2). Comprising five patient scenarios, EMQ demonstrated the highest average word count per item of almost 150. A Tukey post hoc test demonstrated that EMQ were significantly longer compared to SBA, Pick-N, and MTF (Fig. 2A). The MCQs were also significantly different in regard to TTR. EMQ exhibited the lowest degree of lexical variation. The Tukey post hoc tests revealed that TTR values were significantly lower in EMQ than for MTF (Fig. 2B, Table 2). All MCQ item types did not significantly differ from one another with respect to Yule’s K as a measure of the vocabulary richness (Fig. 2C, Table 2).

### Processing time

**Figure 3:**
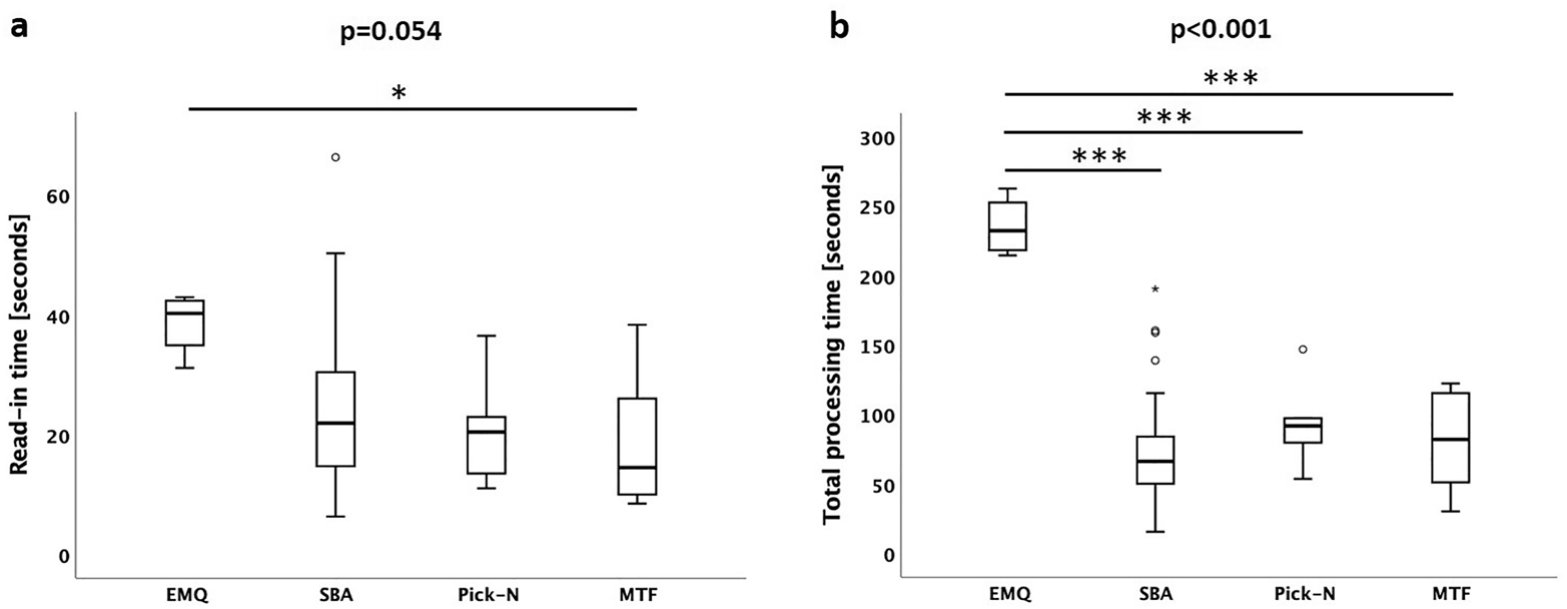
Read-in time and (**a**) total processing time (**b**) for each item type. Parameters are depicted for each item format separately as box plots. *p < 0.05; ***p < 0.001.

Read-in time and total processing time differed across MCQ types, as depicted in Fig. 3. The difference in the read-in time was only significant in the direct comparison of EMQ with MTF, but total processing time was significantly longer in EMQ compared to all other item types.

### Students’ evaluation

The online survey was completed by 77% of the students (119 out of 154). They rated EMQ higher in terms of their clinical relevance and found them more challenging in comparison to established question types (Table 2).

## Discussion

Educators face a number of challenging tasks during the design phase of written examinations to assess student knowledge and abilities fairly. They need to design appropriate items to test the students’ extent of factual knowledge, the ability of students to apply this factual knowledge in the clinical environment, and finally to demonstrate their clinical reasoning and decision-making skills. This study compared EMQ to other established MCQ types (SBA, Pick-N, and MTF). MCQ examinations are traditionally thought to be better at testing knowledge recall^29^ and are usually rather poorly equipped for testing hypothesis-driven reasoning. However, one can put the emphasis on a detailed clinical stem extending beyond the level of factual knowledge. The addition of a scenario fosters the analysis and evaluation of a patient’s symptoms and diagnostics in the context of gender, age, and comorbidities. Here, we elucidate the suitability of EMQ as an assessment method specifically in undergraduate medical education. This particular format requires higher-order cognitive skills to evaluate and balance out information from clinical stems within a specific clinical context. Statistical analyses allow us to shed light on the complex interplay of the word length of MCQ types, readability parameters, and processing time. We also examined the impact of Cronbach's alpha as a measure of internal consistency to calculate the theoretical sample size as an evaluation of resource effectiveness.

### Item analysis

Difficulty, discrimination, and discrimination index are established quality criteria for items in written examinations. In our study, the item difficulty was fairly comparable in all question types; students only scored slightly lower in EMQ. This finding is consistent with the literature^30^ and meets previously published quality criteria^31^. In fact, the difficulty of MCQ is known to increase with the number of response options, irrespective of the question type^32^. The average number of response options for SBA, MTF, and Pick-N was five. Tthe EMQ, however, had eight response options. Nevertheless, the higher number of response options for EMQ did not automatically increase the difficulty in our study.

In contrast, item discrimination and discrimination index were improved in EMQ compared to the other MCQ formats. Thus, for a number of students, the EMQ in the examination proved to be suitable predictors of performance in the whole examination. These results are broadly consistent with earlier research^33^, but differ from more recently published findings^34^. There are several possible explanations for this. Eijsvogels and colleagues applied correction for guessing^35^ on MCQ, but not on EMQ, which could explain the better discriminative capacities of MCQ. Furthermore, all question types in our study were based on clinical practices aimed at testing cognitive abilities and not factual knowledge as it was previously applied during the examinations in first-year medical and biomedical students^34^.

### Readability and processing time

As expected, EMQ comprising five clinical scenarios were longer in text and thus required a longer read-in and total processing time. However, the length of the EMQ did not negatively impact on their readability. It is important to add that TTR indicated an improvement in readability for EMQ compared to the other question types, a parameter which has not been evaluated in any other study so far. Nevertheless, we remain cautious when using TTR, as it is distorted by the length of the text segment^36^, and as text complexity usually decreases with length^23^. This is the reason we added the analysis of Yule’s K, another readability index, which is regarded as invariant for text length^36^. This parameter confirmed that EMQ were indeed not more difficult in terms of readability. A tendency to perform better in MCQ based on preceding longer text passages was previously described by Bae et al. in a non-medical context^37^. Thus, one would expect students to perform even better in EMQ. It is perhaps worth noting that the EMQ in our study were the items with the greatest difficulty. However, the format was associated with better item performance measures including discriminative capacity.

A special focus of our analysis was to determine the ability of EMQ to shorten the duration of examinations, not only in light of the SARS-CoV-2 pandemic. The construct validity of EMQ-based examinations was addressed previously^38^ and led us to focus on reliability in our analysis. Using the Spearman-Brown prediction formula, we estimated that the overall reliability of the examination could be reproduced using as few as ten EMQ items. In doing so, the hypothetical examination could thus be reduced to as little as 40 min in duration, approximately one third of the time required for the original examination. This finding is consistent with that of Swanson and colleagues, who previously demonstrated that EMQ with larger numbers of response options allow for more efficient use of the time available for testing^32^. The purpose of their study was to assess final medical examinations with the focus on diagnostic reasoning, while our questions—irrespective of question type—also included therapeutic considerations. Our data substantiate the potential role of EMQ in the reduction of examination duration, which involves an interesting aspect of reducing contact times during the SARS-CoV-2 pandemic and the potential savings regarding supervisory staff.

### Clinical reasoning

Problem solving as clinical reasoning is one of the major goals to be achieved during medical education, which takes a long period of time (usually at least five to six years)^39^. Irrespective of debates regarding the underlying mechanisms of higher-order thinking, there is no denying the fact that clinical reasoning encompasses cognitive and decision-making processes that enable physicians to choose the most relevant actions in a given context^40^. Medical teaching programs recognize the importance of clinical reasoning and embed its teaching into their curricula^40^. Often used in final medical examinations, EMQ offer the opportunity to monitor clinical reasoning in a way not feasible through traditional MCQ. A previous study, which compared the performance of final-year medical students and fifth-year residents with EMQ, clearly demonstrated the convergent or discriminant validity of the question format. As expected, the latter group performed better in their area of expertise, applying forward reasoning in particular as a typical feature of clinical reasoning^8^. In our study, we did not emphasize any appropriate external criteria to test for clinical reasoning. Nevertheless, it is worthwhile noting that our students recognized the potential of EMQ to address clinical reasoning, as they rated EMQ higher than other MCQ types with respect to their clinical relevance.

To summarize, we were able to confirm that EMQ are an appropriate tool enabling examiners to draw fair conclusions about a student’s ability^33,41^. EMQ were slightly more difficult to score; however item discrimination and discrimination index were clearly higher when compared to the other question types. EMQ were found to have improved readability, although more time was needed to process each single EMQ. Students judged EMQ as clearly challenging, but attributed a significantly greater clinical relevance when compared to the established MCQ formats. Owing to their good reliability, EMQ are resource effective. By economizing on the overall time required for examinations, their incorporation could free up resources potentially required elsewhere.

### Limitations and future perspectives

The strengths of this study lie in the number of students in one semester who were included, as well as the incorporation of EMQ into an already established examination implementing SBA, Pick-N, and MTF-style items. A clear limitation is the fact that we were restricted to one examination. Our data must be interpreted with caution, as only a limited number of item samples besides the SBA and the four EMQ were included in the analysis (five Pick-N and eight MTF). Further studies in end-of-semester examinations during different stages of the curriculum, perhaps even at additional medical schools, will be required to further underline statements on the suitability and statistical features of EMQ. Furthermore, optimization of the examination process should not only focus on its duration, but also on the time needed for preparation. One would expect that EMQ require more preparation time, thus undermining the advantages of shorter examination durations. Moreover, the preparation of EMQ requires experienced educators as well as specific training sessions, whereas the examination itself can simply be accompanied by administrative personnel. But ultimately, the time-saving aspect also affects the students sitting the examination. To investigate this important issue, future research should also address the time needed to create items and assure the quality of different item types.

## Conclusion

We demonstrate that EMQ are feasible, reliable, valid, authentic, and are highly appreciated by undergraduate medical students, who clearly recognized the added value in the examination. As an expression of suitability, EMQ offer high-quality testing with an exceptional degree of clinical relevance. Item analysis was very valuable and students demonstrated how easily they can master the new question format. From the perspective of educators, this study suggests that EMQ are more effective in discriminating good from poor performers in a shorter time frame. Taking into consideration the efficiency of EMQ, faculties should be encouraged to invest in training in constructing good EMQ to offset or even substitute the resources consumed by persisting with established MCQ formats.

## Supplementary Information

Supplementary Information.

## Data Availability

The datasets used and/or analysed during the current study are available from the corresponding author on request.
