# Supplementary material for: Item format statistics and readability of extended matching questions as an effective tool to assess medical students
Source: Sci Rep. 2022 Dec 5;12:20982. doi: 10.1038/s41598-022-25481-y (PMC9723123; doi:10.1038/s41598-022-25481-y)
Supplement: Supplementary file 1 — Supplementary Information. [file 41598_2022_25481_MOESM1_ESM.docx]

**SUPPLEMENTAL MATERIAL**

**Item Format Statistics and Readability of Extended Matching Questions as an Effective Tool to Assess Medical Students**

**Brief title:** EMQ in the Digital Exam for Internal Medicine

Anna Frey^a,b^, MD, Tobias Leutritz^b^, PhD, Joy Backhaus^b^, PhD, Alexander Hörnlein^c^, Sarah König^b^, MD, MME

^a^ Department of Internal Medicine I, University Hospital of Würzburg, Germany

^b^ Institute of Medical Teaching and Medical Education Research, University Hospital of Würzburg, Germany

^c^ University Datacenter, University of Würzburg, Germany

**Contents**

**Supplemental Methods**

EMQ example page 2

**Supplemental Methods**

**EMQ example**

Main complaint: abdominal pain

Option list

(a) Colonic ileus

(b) Acute pancreatitis

(c) Renal colic

(d) Appendicitis

(e) Small bowel ileus

(f) Acute cholecystitis

(g) Ulcerative colitis

(h) Duodenal ulcer

Lead-in:

Please match the above response options to the clinical scenarios given below. Each response can only be chosen once.

Item stem:

1. A 45-year-old man with a history of gallstones presents to the emergency department with massive epigastric pain radiating round to the back and accompanied by persistent vomiting.

2. A 28-year-old man complains of stabbing spasmodic pain in the left side and upper left abdomen. The pain come in waves and radiates to the groin.

3. A 44-year-old woman has persistent pain in the upper right abdomen accompanied by vomiting and fever. Murphy's sign is positive.

4. A 26-year-old man with a history of abdominal surgery complains of colicky periumbilical pain accompanied by bilious vomiting spells that come immediately after every episode of pain.

5. A 50-year-old man complains of epigastric pain that increases at night and subsides during the day after food or milk intake.

Answers/correct combinations:

1 – B

2 – C

3 – F

4 – E

5 – H
